# A Balancing Act: Navigating Hypertensive Disorders of Pregnancy at Very Advanced Maternal Age, from Preconception to Postpartum

**DOI:** 10.3390/jcm12144701

**Published:** 2023-07-15

**Authors:** Miriam Lopian, Lior Kashani-Ligumsky, Ariel Many

**Affiliations:** 1 Department of Obstetrics and Gynecology, Mayanei Hayeshua Medical Center, Bnei Brak 51544, Israel; liorkashani@yahoo.com (L.K.-L.); many@tauex.tau.ac.il (A.M.); 2 Sackler School of Medicine, Tel Aviv University, Tel Aviv 69978, Israel

**Keywords:** advanced maternal age, hypertension, pre-eclampsia

## Abstract

The decision to postpone parenting has gained momentum in recent years, a shift driven by evolving social dynamics and improved access to fertility treatments. Despite their increasing prevalence, pregnancies at advanced maternal ages are associated with increased risks of adverse maternal and neonatal outcomes. This article addresses the association between advanced maternal age and hypertensive disorders of pregnancies (HDPs), which are more prevalent and a significant cause of maternal morbidity and mortality in this population. This review explores the biological mechanisms and age-related risk factors that underpin this increased susceptibility and offers practical management strategies that can be implemented prior to, as well as during, each stage of pregnancy to mitigate the incidence and severity of HDPs in this group. Lastly, this review acknowledges both the short-term and long-term postpartum implications of HDPs in women of advanced maternal age.

## 1. Introduction

The decision to postpone parenthood has become increasingly prevalent and has subsequently resulted in a shift from the traditional childbearing years. This trend has been largely driven by social and cultural advancements in women’s rights, improved access to contraception and legalized abortion. In addition, significant advances in artificial reproductive technology (ART) have dramatically improved the fertility rates of women of advanced maternal age and, to some extent, have facilitated deferred parenthood. Between 2018 and 2020, 3.5% of live births were to women over the age of 40; this was an increase of 2.4% from 2000 to 2002, a trend which is anticipated to continue [1]. The term advanced maternal age (AMA) is commonly used to describe women aged 35 or older at the time of delivery [2]. This age cutoff was established historically based on reduced fertility and the increased risks of chromosomal abnormalities and pregnancy loss that occur after this age. However, advances in ART, such as oocyte cryopreservation and donation, have made it possible for women to have children later in life, even into their fifth and sixth decades. As a result, the terms “very advanced maternal age (VAMA)” and “extremely advanced maternal age (EAMA)” have emerged, referring to women who give birth at the ages of 40 and 45 years or older, respectively [3].

Delaying childbearing until later in life can carry significant risks for women and their babies. Women who are older than 35 at delivery have increased rates of gestational diabetes [4], preterm birth [5], stillbirth [6], cesarean delivery [7], pre-eclampsia [8] and maternal and neonatal morbidity and mortality [8]. These risks become even more pronounced with further increases in maternal age. In fact, a recent report from the Centers for Disease Control cited maternal mortality rates of 107.8/100,000 in women over 40 compared with 22.8/100,000 for women aged 25–39 [9]. One particularly strong association is between advancing maternal age and the risk of developing hypertensive disorders of pregnancy (HDPs), which includes gestational hypertension, chronic hypertension, pre-eclampsia–eclampsia and Hemolysis, Elevated Liver Enzymes and Low Platelets (HELLP) Syndrome [10]. Although HDPs complicate 2–8% of pregnancies overall [11], they affect 18% of pregnancies in women aged 35–44 and 35% in women over 45 [12]. More importantly, though, HDPs are responsible for up to 30% of maternal mortality because of stroke, eclampsia, disseminated intravascular coagulopathy and renal failure [12].

As the trend of delaying motherhood continues to grow globally, the prevalence of HDPs is expected to rise, and with it, a significant health burden. That said, many of the complications associated with HDPs can be prevented, highlighting the importance of providing expectant mothers with close follow-up and timely intervention to optimize both maternal and neonatal outcomes in these pregnancies.

This review will shed light on the factors contributing to the increased risk of HDPs in women of very advanced maternal age (>40 years). Furthermore, it will provide practical management guidelines that can be implemented throughout all stages of pregnancy to reduce the incidence and severity of HDPs in these women.

## 2. Part I: Understanding the Relationship between Maternal Age and Hypertension-Related Pregnancy Complications—Theoretical Perspectives

### 2.1. Biological Mechanisms Underlying Pre-Eclampsia

Pre-eclampsia is defined by the International Society for the Study of Hypertension in Pregnancy (ISSHP) as gestational hypertension accompanied by one or more of the following new-onset conditions at ≥20 weeks’ gestation. These conditions include proteinuria, evidence of maternal end-organ dysfunction (neurological complications, acute kidney injury, hematological complications, or liver involvement) and evidence of uteroplacental malperfusion (e.g., placental abruption, angiogenic imbalance, fetal growth restriction, abnormal umbilical artery, Doppler waveform analysis or intrauterine fetal death) [13]. Although many risk factors for HDPs have been described in the literature, the exact causes and mechanisms of pre-eclampsia remain the subject of much debate. Many hypotheses about the biological processes involved in pre-eclampsia have been proposed, but no single theory can fully account for every instance of this condition. As a result, it is plausible that pre-eclampsia encompasses a variety of conditions, each distinguished by its own distinct underlying physiological process.

The most cited theory is that of abnormal placentation. According to this theory, defective placentation occurs because of suboptimal remodeling and the transformation of maternal spiral arterioles by migrating trophoblasts. This results in placental hypoperfusion and, consequently, as hypoxia and ischemic injury. Ischemia triggers the release of anti-angiogenic factors, e.g., soluble fms-like tyrosine kinase-1(sFlt-1) into the maternal circulation, which results in widespread endothelial dysfunction and inflammation, resulting in hypertension and organ dysfunction such as proteinuria. The strongest evidence for this theory is that pre-eclampsia is cured within days of delivery of the placenta [14,15].

An alternative hypothesis suggests that pre-eclampsia is a manifestation of subclinical maternal cardiovascular dysfunction [16]. According to this theory, cardiac dysfunction results in uteroplacental hypoperfusion, resulting in placental dysfunction and the subsequent development of pre-eclampsia. Evidence for this theory comes from the fact that patients with congenital heart disease are at increased risk of pre-eclampsia and growth restriction [17]; the same is true for patients who have poor pre-pregnancy hemodynamic profiles, namely, higher systemic vascular resistance and mean arterial pressure [18].

Another theory highlights the role of immunological factors and suggests that altered maternal immune responses to trophoblast invasion results in defective placentation, hypoxia and angiogenic imbalance. Support for this theory comes from evidence of higher rates of pre-eclampsia in pregnancies conceived via oocyte donation [19] (as well as those with less exposure to paternal antigens (ICSI, nulliparous, barrier contraception) [20]. Other theories have been proposed. However, a full discussion about the pathophysiology of pre-eclampsia is beyond the scope of this work.

### 2.2. The Association between Advanced Maternal Age and Hypertensive Disorders of Pregnancy

Although there is strong evidence linking advanced maternal age to the development of hypertensive disorders during pregnancy, reports on the risk of gestational hypertension in this population are conflicting. While some studies report a 60% increased risk of gestational hypertension in women over the age of 40 [21,22], others have found no significant difference in risk compared to younger women [23,24]

In contrast, there is clear evidence for an increased risk of developing pre-eclampsia in women of advanced maternal age. Poon et al. demonstrated that this risk starts to increase as early as 32 years of age and rises by approximately 4% a year [25]. Similarly, Khalil et al. reported that compared with women under the age of 35, the risk of developing pre-eclampsia was 8% higher in women aged 35–39 and 50% higher in women aged 40 and older. Furthermore, this association persisted after adjusting for confounding variables such as BMI, chronic hypertension and maternal diabetes, as well as obstetric and smoking history [23].

Recent evidence suggests that these risks continue to increase after the age of 40. According to a meta-analysis of more than 10 million births, women over the age of 35 have a relative risk of 1.2 for developing pre-eclampsia. This doubles to 2.4 for women over the age of 40 and triples to 3.6 in women over 45 [26]. Similarly, using a reference group of women aged 40–45, Smithson et al. reported a twofold increase in the rates of pre-eclampsia–eclampsia in women older than 45 [27] and, more recently, Schwartz et al. reported that women over the age of 50 had double the risk of developing pre-eclampsia compared with women aged 40–49 [28].

### 2.3. What Factors Drive this Increased Risk?

Although there is much evidence demonstrating that older women are at higher risk of HDPs, there is little understanding of the biological mechanisms underlying this relationship and it is unclear whether age exerts its effect independently or whether other confounding factors, such as pre-existing comorbidities or the use of artificial reproductive technology (ART), are responsible for this association.

It is certainly biologically plausible that advanced maternal age is an independent risk factor for pre-eclampsia. One theory suggests that pre-eclampsia occurs when maternal cardiovascular adaptation to pregnancy is disrupted [29]. Indeed, the normal aging process is associated, amongst other changes, with decreased vascular compliance and endothelial dysfunction [30], which, in theory, can hamper these physiological cardiovascular adaptations to pregnancy. Studies have shown that older women have increased impedance in the uterine artery during the first trimester [31], reduced spiral vasculature volume [32] and elevated levels of anti-angiogenic factors, such as sFLT-1 [33], all factors associated with compromised placental function. In addition, the increased risk of other placentally mediated conditions such as growth restriction [34] and stillbirth [6] in older women provides additional evidence for impaired placentation associated with advanced maternal age.

Other mechanisms proposed to explain an independent effect of maternal aging include a raised inflammatory state [35] as well as a decline in circulating estradiol levels [36] in older women, both factors that have been implicated in the pathophysiology of pre-eclampsia.

A recent study demonstrated that women with diminished ovarian reserve (DOR) are at a significantly increased risk of developing pre-eclampsia (aOR 3.05) compared with women without DOR [37]. Low ovarian reserve is known to be associated with increased cardiovascular morbidity later in life [38], and the results of this study strengthen the association between ovarian aging, cardiovascular morbidity, and the risk of developing pre-eclampsia.

Although it is plausible that normal aging is responsible for some of the elevated risk of developing HDPs in this population, the increasing prevalence of other risk factors associated with HDPs that accrue with advancing age suggests that age does not exert its effect alone.

### 2.4. The Impact of Advanced Reproductive Technology on the Development of Hypertensive Disorders of Pregnancy

Pregnancies conceived using ART are at an almost 50% increased risk of being complicated by HDPs; this remains true even after adjusting for confounders such as age, parity, plurality, and comorbidities [39]. Pathophysiological mechanisms implicated in the elevated risk of hypertensive diseases in ART pregnancies involve defective trophoblast invasion owing to the influence of ovarian stimulation on the endometrium as well the formation of the chorion in vitro [40]. There are also emerging data that cycle characteristics significantly modify this risk. Pregnancies in women over 45 are rarely spontaneous, and while assisted reproductive technology can improve live-birth rates in younger, infertile women, it has little benefit in much older women. Therefore, a significant proportion of pregnancies at this age are achieved using donor eggs. Pregnancies achieved using donor oocytes have a 5–7-fold increased risk of being complicated by pre-eclampsia [19]. This phenomenon has been attributed to increased immune activation from the foreign oocyte, resulting in defective placentation and, ultimately, pre-eclampsia [41]. In addition, protocols involving ovarian hyperstimulation, which result in supraphysiological levels of circulating estradiol, are also associated with a higher risk of developing HDPs [42]. Lastly, pregnancies achieved from frozen rather than fresh embryo transfer are 1.7 times more likely to be complicated by pre-eclampsia [43]. Apart from immune-related processes, another theory explaining the higher risk of hypertensive disorders of pregnancy (HDPs) in individuals undergoing oocyte donation or frozen embryo transfer relates to the absence of the corpus luteum in these cycles. This absence could lead to changes in the hormonal environment and the makeup of vessel-active substances like Relaxin. The deficiency of these elements might obstruct the cardiovascular system’s adaptation to pregnancy during the first trimester [44].

### 2.5. Is the Increased Risk of Hypertensive Disorders of Pregnancy in Older Women Exacerbated by the Increased Prevalence of Multiple Gestations in This Population?

To improve IVF outcomes, patients of advanced maternal age often undergo the transfer of more than one embryo. This practice has resulted in a higher prevalence of multiple pregnancies in these patients, particularly when donor eggs, which have a higher implantation potential, are used [45]. Indeed, in 2018, the twin birth rate in the United States for patients over the age of 40 was 51/1000, compared with 17/1000 in patients under the age of 20 [46]. Multiple gestations are a known risk factor for the development of HDPs. Indeed, the risk of developing an HDP is proportional to the number of fetuses in the pregnancy [47]. Mechanisms proposed to explain this association include the greater hemodynamic demands on the cardiovascular system in multiple gestations that occur because of greater placental mass [48]. It is therefore possible that the presence of multiple gestations in these patients contributes to the elevated risk of pre-eclampsia in this population.

### 2.6. The Contribution of Accrued Chronic Diseases with Advancing Maternal Age on the Risk of HDPs

As individuals age, chronic diseases accrue. Several maternal pre-existing comorbidities have been found to increase the risk for the development of hypertensive disorders of pregnancy. As an example, maternal obesity, which has significant effects on various health systems, has also been shown to have a dose-dependent effect on the risk of developing pre-eclampsia. One study demonstrated that patients with a BMI greater than 26 kg/m^2^ have twice the risk of pre-eclampsia, whilst those with a BMI greater than 30 kg/m^2^ have three times the risk [49]. Another significant factor is the presence of chronic hypertension, which is more prevalent in older patients and increases the risk of developing pre-eclampsia up to five times [50]. Other disorders that are high-risk factors for the development of hypertensive disorders of pregnancy include renal diseases and autoimmune diseases such as systemic lupus erythematosus, anti-phospholipid syndrome and pre-existing diabetes mellitus [2], all of which are more prevalent in older mothers.

Whilst there is evidence indicating that maternal age is an independent risk factor for the development of hypertensive disorders of pregnancy [23], other studies suggest that this risk may be mediated through the increased prevalence of multiple comorbidities or the need for assisted reproduction. Additionally, some studies show that after controlling for confounding variables, the risk of developing HDPs is not greater in women of very advanced maternal age [24,51]. Nonetheless, we believe that regardless of mechanism, patients of very advanced maternal age merit special attention before and during pregnancy to optimize obstetric outcomes.

## 3. Part II: Preconception, Antepartum and Postpartum Clinical Considerations for the Prevention and Management of Hypertensive Disorders of Pregnancy in Women of Very Advanced Maternal Age

### 3.1. Preconception Counseling—A Window of Opportunity

The age-old adage “an ounce of prevention is worth a pound of cure” rings particularly true for women of very advanced maternal age who are contemplating pregnancy. The importance of preconception counseling for these women cannot be stressed enough. Meticulous planning and comprehensive management during this period can significantly improve pregnancy outcomes. Fortunately, due to a reliance on ART in this population, these pregnancies are usually planned, affording obstetricians the opportunity to have a candid discussion with the patient about the risks associated with pregnancy at this age whilst considering their individualized risk factors.

Approximately half of women over the age of 45 have two or more chronic diseases [52]. Therefore, it is crucial to take a thorough medical, obstetric and social history, as well as perform a comprehensive physical examination and a set of investigations to screen for risk factors associated with HDPs. While there are no specific evidence-based guidelines on which investigations are necessary, we recommend obtaining the following preliminary investigations at a minimum.

This initial assessment serves multiple purposes. Firstly, it screens for any latent medical conditions more common in this age group that could influence the outcome of pregnancy or be influenced by it (Table 1).

Secondly, for patients with known co-existing conditions, this assessment gauges the extent of disease control prior to conception. This information allows for personalized counseling regarding their unique pregnancy-related risks, the significance of maintaining disease control before and during pregnancy and, if relevant, the potential benefits of aspirin treatment during pregnancy, a topic explored in further detail later. It is essential to note that patients with newly diagnosed or poorly controlled diseases are at the highest risk for adverse outcomes during pregnancy. Therefore, efforts should be taken in the preconception period to achieve optimal disease control, thereby enhancing pregnancy outcomes [49,50].

Post-assessment, patients can be classified into low-risk and high-risk categories, enabling appropriate counseling and management. For older patients who are free of chronic diseases or other risk factors, reassurance can be provided that, despite their age, their pregnancy and childbirth are likely to be uneventful [24,51]. Lastly, this preconception evaluation provides a valuable record of the patient’s baseline laboratory results. These will serve as a crucial point of reference later in pregnancy should questions arise concerning the significance of specific findings during evaluation. For instance, knowing the baseline level of urinary protein excretion might be crucial in confirming or refuting a diagnosis of pre-eclampsia during pregnancy.

### 3.2. Strategies for Mitigating the Risk of Adverse Outcomes during Pregnancy

Lifestyle changes such as weight loss, daily exercise and, if relevant, stopping smoking can help to reduce the risk of developing HDPs [53]. Additionally, strong consideration should be given to avoiding multiple-embryo transfers, particularly when donor oocytes are being used. Performing a multiple-embryo transfer significantly increases the chances of conceiving a multiple pregnancy, which is an independent risk factor for the development of hypertensive disorders of pregnancy [47]. There is also evidence that patients older than 45 carrying a multiple pregnancy are particularly vulnerable to developing pre-eclampsia and are more likely to be admitted to intensive care compared with younger patients carrying twin pregnancies [54].

Most importantly, attention should be directed towards optimizing control of pre-existing diseases, since this is the primary determinant of pregnancy complications. While this may necessitate postponing attempts at conception for a few months, a recommendation that may be met with resistance, especially by those for whom oocyte donation is not an option, the importance of optimizing chronic conditions cannot be overstated as it is arguably the single most effective way to mitigate adverse maternal outcomes. This information should be communicated to the patient in a clear and sensitive manner to encourage compliance.

Chronic hypertension is the strongest risk factor for the development of pre-eclampsia. Its prevalence increases steadily with age, such that 1 in 3 women over the age of 40 suffer from chronic hypertension compared with only 1 in 14 women under 40 years of age [12].

Chronic hypertension has traditionally been defined as a systolic or diastolic blood pressure ≥ 140 mm Hg or ≥90 mm Hg, respectively, prior to the onset of pregnancy. However, more recent guidelines refer to a systolic blood pressure of 120–129 mmHg and diastolic pressure less than 80 mmHg as elevated blood pressure, and Stage 1 hypertension is defined as a systolic blood pressure of 130–139 mmHg and diastolic blood pressure between 80 and 89 mmHg [55]. There are data delineating a dose–response relationship between blood pressure and pre-eclampsia risk. This risk starts to increase with blood pressures in the “elevated” range and is 6-fold higher in patients with Stage 1 hypertension [55].

Until recently, the initiation of antihypertensive medication during pregnancy was advised only in patients with systolic or diastolic blood pressures greater than or equal to 160 mmHg or 110 mmHg, respectively [55]. This advice was partly due to theoretical concerns of fetal growth restriction owing to a relative reduction in uteroplacental blood flow as well as little evidence of maternal benefit with tighter blood pressure control [56]. However, the recent results of the CHAP (Control of Hypertension in Pregnancy) trial have highlighted the benefits of tighter blood pressure control. This randomized control trial demonstrated a 20% reduction in the risk of developing pre-eclampsia in patients with chronic hypertension receiving intensive blood-pressure management during pregnancy, i.e., treatment of systolic or diastolic blood pressure greater than 140 mmHg and 90 mmHg, respectively, compared with patients in whom antihypertensive therapy was initiated when systolic or diastolic blood pressure reached levels greater than 160 mmHg and 105 mmHg, respectively [57]. As such, the ACOG has updated their guidance and advised utilizing 140/90 as the threshold for commencing or titrating antihypertensive treatment during pregnancy, rather than 160/110 mmHg [58].

As for patients with Stage 1 hypertension (130–139/80–89 mmHg), who are recognized to have a 6-fold increased risk of developing HDPs [55], the clinical utility as well as the cost-effectiveness of treatment with the aim of reducing blood pressure to this extent (130–139/80–89 mmHg) have not been studied. It remains uncertain whether this would result in a further reduction in the risks of developing pre-eclampsia.

### 3.3. Therapeutic Options

Despite being responsible for significant maternal and neonatal morbidity and mortality, there is currently no effective treatment for pre-eclampsia other than delivery of the fetus and placenta, which is often associated with significant neonatal morbidity because of iatrogenic prematurity. As a result, most therapeutic interventions that have been trialed involved attempts at pre-eclampsia prophylaxis rather than treatment. Numerous preventive strategies have been trials. However, as of now, no single superior option can prevent all cases of pre-eclampsia. This section will give a brief review of the most promising treatments to date.

### 3.4. Low-Dose Aspirin

Beyond its well-known analgesic properties, aspirin has cardioprotective effects. The rational for its use in preventing pre-eclampsia comes from the observation that, in pre-eclampsia, there are high levels of platelet-derived thromboxane production. Aspirin, when used at low doses, effectively inhibits the synthesis of platelet-derived thromboxane [59].

Indeed, the ASPRE trial demonstrated that the administration of low-dose daily Aspirin reduces the rates of preterm pre-eclampsia by 62% in patients considered high-risk for the disease [60]. Results from a recent meta-analysis also report reduced rates of perinatal mortality, preterm birth, and small-for-gestational-age neonates with Aspirin prophylaxis during pregnancy [61]. Furthermore, the U.S Services Preventive Task Force guidelines recommend initiating daily Aspirin at 12–16 weeks in patients with one or more high-risk factors or two or more moderate-risk factors [62]. According to these guidelines, patients over 35 are at moderate risk and require the presence of another risk factor to be given Aspirin during pregnancy. Based on the evidence of a dose–response relationship between age and the risk of developing pre-eclampsia [23,25], we recommend initiating Aspirin treatment at 12 weeks of gestation in patients over the age of 45 even in the absence of other risk factors.

### 3.5. Calcium Supplementation

A large meta-analysis showed a 50% reduction in the risk of pre-eclampsia in patients who received calcium supplementation during pregnancy. However, this was only true for patients with low calcium intake [63].

### 3.6. Metformin

Metformin, an oral anti-glycemic agent used to manage Type 2 Diabetes, Gestational Diabetes and Polycystic Ovary Syndrome, acts by inhibiting gluconeogenesis and enhancing insulin sensitivity in peripheral tissues. Moreover, evidence from human studies suggests that Metformin can reduce the secretion of the antiangiogenic markers that are implicated in the pathophysiology of pre-eclampsia, namely, soluble fms-like tyrosine kinase 1 (sFlt-1) and soluble endoglin (sEng). This study demonstrated that Metformin also reduced endothelial dysfunction and promoted both angiogenesis and vasodilation [64].

Several studies, including systematic reviews and meta-analyses, have explored Metformin’s potential role in mitigating pre-eclampsia. However, no clear consensus has been reached regarding its impact on the development of this condition. While some studies suggest a reduced rate of pre-eclampsia [65,66,67], others have found no such effect [68,69,70].

These conflicting data might be the result of Metformin being effective only in specific patient subgroups, or only at certain doses or gestational ages. Even if Metformin does not prevent pre-eclampsia outright, its potential ability to decrease antiangiogenic factors may, in theory, lessen the severity or delay the deterioration of this pre-eclampsia. Interestingly, the results of a recent phase II double-blind randomized controlled trial, in which 3 g of oral Metformin was administered daily in patients with severe preterm pre-eclampsia, demonstrated a significant prolongation of gestation (mean 11.5 days) in patients treated with Metformin compared with placebo [71]. Additional studies are required to elucidate the optimal population who could benefit from Metformin for pre-eclampsia prophylaxis.

### 3.7. Statins

Another drug class that has gained interest due its potential role in reducing the incidence of pre-eclampsia is statins. Statins are traditionally used to lower cholesterol. Their potential role in reducing the risk of developing pre-eclampsia comes from studies demonstrating a protective effect of Pravastatin on endothelial cells as well as preventing the release pf anti-angiogenic factors, namely sflt-1/sEng, which are known to play a significant role in the pathogenesis of pre-eclampsia [72]. Several studies have shown reductions in the rate of pre-eclampsia in high-risk patients taking Statins during pregnancy, alone or in addition to Aspirin [73,74]. A recent RCT evaluating the effects of Pravastatin in women at a high risk for developing pre-eclampsia reported half the rate of pre-eclampsia in patients receiving Pravastatin (17.5% vs. 35%) compared with placebo. Although this result did not reach statistical significance, they did demonstrate a significant reduction in the rates of preterm delivery as well as composite neonatal morbidity in this group. Furthermore, patients receiving Pravastatin treatment showed no deterioration in circulating anti-angiogenic markers, whilst those receiving the placebo had a significant deterioration in these factors. This finding suggests that the improved maternal and neonatal outcomes in patients receiving Pravastatin prophylaxis may be mediated by preventing angiogenic imbalance [75]. Further research is required to determine whether there is a role for Pravastatin in secondary prophylaxis of pre-eclampsia.

### 3.8. Surveillance of Pregnancies in Women of Very Advanced Maternal Age

Pregnancies at very advanced maternal age require close follow-up during pregnancy and an emphasis on regular screening for the development of HDPs. We record blood pressure and screen for proteinuria prior to conception, twice prior to 20 weeks of gestation, every 4 weeks from 20 weeks and then every fortnight from 32 weeks until term. In addition, we obtain a complete blood count as well as liver and renal profile at 6–10 weeks, 24–28 weeks and again at 32–34 weeks of gestation.

Pregnancies in individuals of very advanced maternal age are at increased risk of both intrauterine fetal death as well as fetal growth restriction [23,34]. Although there is little evidence supporting this, in line with the advice of the Society for Maternal and Fetal Medicine/ACOG guidelines on pregnancy at advanced maternal age [2], we initiate antenatal testing including fetal growth assessment at 32 weeks of gestation, the timing and frequency of which should be individualized according to other risk factors such as the presence of pre-existing diabetes, renal disease and chronic hypertension.

Despite the implementation of strategies to reduce the risk of developing pre-eclampsia, 6–10% of individuals over the age of 45 will develop a hypertensive disorder of pregnancy [76,77]. Whilst many studies have demonstrated an increased prevalence of pre-eclampsia with advancing age, few have addressed the effect of age on the manifestation of pre-eclampsia. In a secondary analysis of the HYPITAT-I, van der Tukk et al. demonstrate that older patients who develop hypertensive disorders of pregnancy are at an increased risk for more severe manifestations of pre-eclampsia compared with younger patients [78], with studies reporting an increased tendency to develop pulmonary oedema, hemodynamic compromise [79], severe postpartum hypertension, elevated creatinine [80] and peripartum cardiomyopathy [81] in older patients.

Women of very advanced maternal age who develop pre-eclampsia undergo significant hemodynamic alterations, including age-related increases in vascular resistance, endothelial dysfunction and reduced cardiovascular reserve, which are then complicated by the widespread vasospasm and intravascular depletion associated with pre-eclampsia [82]. These manifestations may be further exacerbated in patients who have pre-existing diseases [83] or are obese [84]. In addition, we, therefore, recommend a cautious approach towards any interventions that may further increase blood pressure. These include, but are not limited to, excessive fluid administration during labor and delivery [85] as well as the use of non-steroidal anti-inflammatory drugs, which are known to elevate blood pressure by causing vasoconstriction as well as sodium and water retention [86].

Specific mention should be made regarding the choice of anti-hypertensive medication in this population. There is evidence suggesting that age may affect the response to a specific anti-hypertensive agent. Stott et al. [87] demonstrated that the pathophysiology resulting in hypertension in older women differs to that in younger women. Hypertension in older mothers is more often a result of a high resistance, low output cardiovascular state. This is in comparison to younger patients, who have a “hyper-dynamic” profile to their hypertension, as reflected by a higher cardiac output and stroke volume and normal vascular resistance. Older patients who are more likely to have a vasoconstrictive profile to their hypertension are more often resistant to treatment with labetalol (beta blockers) and have been shown to respond better to vasodilator therapy such as calcium channel blockers. Indeed, the Society of Obstetrics and Gynecology Canadas’ guidelines on hypertension in pregnancy advise that patient characteristics including hemodynamic profile should be used to guide the choice of anti-hypertensive medication [88].

### 3.9. The Role of Circulating Angiogenic and Antiangiogenic Factors in the Management of Pregnancies at Risk of Developing Pre-Eclampsia

The discovery by Levine et al., that women with pre-eclampsia have elevated circulating levels of antiangiogenic factors—namely, soluble fms-like tyrosinekinase-1 (sFlt-1)—and lower levels of pro-angiogenic factors—namely, vascular endothelial growth factor (VEGF) and placental growth factor (PlGF)—proportional to the severity of pre-eclampsia [88], has led to the development of clinical assays measuring the sFlt-1/PlGF ratio, which, at specific cutoffs, can predict, diagnose and rule-out pre-eclampsia [89,90,91] as well as predict the development of adverse outcomes related to pre-eclampsia and the time to delivery [92]. Indeed, several European countries have incorporated the measurement of the sFlt-1/PlGF ratio into clinical use [92]. However, these tests have not yet been proven to be stand-alone diagnostic tests for pre-eclampsia, but rather should be used in conjunction with other clinical parameters for evaluation.

These assays have added value for patients of very advanced maternal age for several reasons. Older patients are more likely to have comorbidities such as chronic hypertension, chronic kidney disease (CKD), systemic lupus erythematosus (SLE) and diabetes, which not only increases the risk of developing pre-eclampsia but can also contribute to diagnostic uncertainty due to similarities in clinical presentations between exacerbation of the underlying condition and pre-eclampsia. An accurate diagnosis is crucial to determine optimal management, which may include delivery, often in the preterm period.

The sFlt-1/PlGF ratio has been shown to accurately discriminate between CKD [93], APLA and SLE [94]; pre-existing diabetes [95]; and pre-eclampsia and, therefore, prevent iatrogenic preterm delivery in cases where PET is ruled out. In addition to its use in symptomatic patients, the incorporation of angiogenic factors to first-trimester prediction models of pre-eclampsia in low-risk patients substantially improves predictive accuracy [96].

In high-risk patients, including women over the age of 40, monthly sFlt-1/PlGF ratio measurements in asymptomatic patients can help determine the frequency and intensity of antenatal surveillance in patients at risk as well as reassure those patients considered not to be at risk.

Lastly, a recent consensus statement [97] suggests that, at term, the sFlt-1/PlGF ratio can help determine the need for delivery. The ROBUST study demonstrated that low sFlt-1/PlGF ratios at term are associated with a low risk of adverse pre-eclampsia-related outcomes and can therefore be managed expectantly, whilst delivery should be considered in those with higher ratios since they are at a higher risk of adverse outcomes [98]. That said, it is important to note that there are no studies suggesting that this ratio can serve as a stand-alone test for determining the timing of delivery in patients at risk of pre-eclampsia [97]. Instead, it should be used in conjunction with traditional markers of disease, such as blood pressure, ultrasound findings and laboratory evaluations.

### 3.10. Timing of Delivery

When pregnancies are complicated by hypertensive disorders, it is often necessary to proceed with an indicated delivery. Decisions regarding the optimal timing of delivery are made after careful consideration of the potential maternal and neonatal risks. On the one hand, there is a risk of maternal deterioration and intra-uterine fetal death if the pregnancy is continued. On the other hand, there is a risk of neonatal morbidity and mortality associated with preterm delivery. The ACOG recommends delivery at 37+0 weeks in patients with gestational hypertension or pre-eclampsia without severe features [99]. This recommendation is based on data from HYPITAT-1 [100] which demonstrated improved maternal outcomes with the induction of labor at 37 weeks for patients with mild hypertensive disease. However, the mean maternal age in the HYPITAT-I trial was 30.4 years. In patients over the age of 45, the risk of both maternal deterioration as well as the background risk of intrauterine fetal demise is greater than that in younger patients [23,34,62]. This raises questions regarding the applicability of data from HYPITAT-1 in this population, and more studies are needed to determine the optimal timing of delivery in patients with pre-eclampsia who are at a high risk of deterioration, either due to very advanced maternal age or comorbidities. Meanwhile, decisions regarding the timing of delivery in these patients should be individualized and take into consideration the increased risk of maternal deterioration as well as the increased respiratory morbidity associated with delivery in the late preterm period. It is our opinion that consideration should be given to lowering the gestational age threshold for delivery or at least increasing the intensity of antenatal maternal surveillance in women over the age of 45 whose pregnancies are complicated by gestational hypertension or pre-eclampsia without severe features.

### 3.11. Post-Partum Considerations

It is crucial to closely monitor patients who have experienced HDPs during the postpartum period. Although delivery is traditionally considered the cure for pre-eclampsia, shifts of extracellular fluid back into the vascular space, amongst other factors, may cause a temporary rise in blood pressure between the third and sixth postpartum day [83,84]. As older women face higher risks [80], we advise against discharge until blood pressure has stabilized for at least 24–48 h. Following discharge, it is prudent to increase the frequency of blood-pressure surveillance, especially in the first week postpartum, during which blood pressure can often remain high or even worsen [83,84]. We advise home-monitoring of blood pressure daily in the first week following discharge. After that, once-weekly blood pressure monitoring is sufficient until the patient has attended her 6-week checkup. This is essential to screen for the development of de novo post-partum hypertension or pre-eclampsia as well as unmask the presence of previously undiagnosed chronic hypertension. If hypertension persists after 6 weeks post-partum or is resistant to maximal doses of antihypertensive therapy, a thorough workup should be conducted to exclude any secondary causes of hypertension [83].

Patients who experience hypertensive disorders of pregnancy have higher lifetime risks of developing cardiovascular and cerebrovascular disease including heart failure, myocardial infarction and chronic hypertension and stroke, as well as all-cause mortality [100]. In addition, there is evidence from a secondary analysis of The Women’s Health Initiative Study that women who deliver at a very advanced maternal age also have an elevated risk of developing stroke, myocardial infarction, and cardiovascular mortality [101] There are currently no available data on the long-term cardiovascular and cerebrovascular outcomes in patients who experienced a hypertensive disorder during pregnancy at a very advanced maternal age. It is certainly feasible that these two risk factors could act synergistically, increasing the risk of these complications further. Therefore, it is crucial to counsel all affected patients regarding their elevated cardiovascular risk and encourage them to modify other cardiovascular risk factors that they may have, such as obesity, smoking, hypercholesterolemia, and chronic hypertension. Furthermore, a plan should be instituted to increase cardiovascular surveillance to monitor and manage any potential complications.

## 4. Summary

Compared with their younger counterparts, patients who are pregnant at a very advanced maternal age are at a higher risk of developing hypertensive disorders of pregnancy as well as experiencing severe complications of these diseases. While part of this risk is attributed to maternal age itself, other age-related risk factors, particularly the use of ART and the presence of associated comorbidities, further compound this risk. Although some of these risk factors are modifiable, age itself is not, and this needs to be communicated to the patient in an honest and sensitive way. Efforts should be directed towards mitigating any residual risk to the patient. In the preconception period, these measures include avoiding the transfer of multiple embryos, frozen embryo transfers, encouraging exercise and weight loss and optimizing chronic medical conditions. During pregnancy, daily Aspirin treatment is advised from 12 to 16 weeks of gestation to reduce the risk of preterm pre-eclampsia. In addition, heightened maternal and fetal surveillance is warranted and involves frequent blood-pressure screening as well as antenatal testing and, possibly, early delivery to optimize maternal and neonatal outcomes in this at-risk population. This surveillance should extend into the immediate postpartum period to screen for the development of postpartum pre-eclampsia or the unmasking of undiagnosed chronic hypertension. Lastly, in the long term, patients with pregnancies that are complicated by pre-eclampsia should be informed of their residual cardiovascular risk and enter long-term cardiac surveillance.

## Figures and Tables

**Table 1:** Recommended investigations prior to pregnancy in women of very advanced maternal age.

General	Laboratory Investigations	Imaging
Body mass index	Complete blood count	Electrocardiogram
Blood pressure	Liver function tests	Echocardiogram (in patients with longstanding hypertension, abnormal ECG or a cardiac murmur)
Heart rate	Calcium and vitamin D	Chest X-ray
Urine screening for proteinuria	Renal profile	
	Thyroid function tests	
	Lipid profile	
	Fasting glucose	
	HbA1c (in patients with pre-existing diabetes)	

## References

[1] https://www.marchofdimes.org/peristats/data?reg=99&top=2&stop=5&lev=1&slev=1&obj=3.

[2] Gantt A., Metz T.D., Kuller J.A., Louis J.M., Cahill A.G., Turrentine M.A., American College of Obstetricians and Gynecologists’ Committee on Clinical Consensus-Obstetrics, Society for Maternal-Fetal Medicine, American College of Obstetricians and Gynecologists (2023). Obstetric Care Consensus #11, Pregnancy at age 35 years or older. Am. J. Obstet. Gynecol..

[3] Lean S.C., Derricott H., Jones R.L., Heazell A.E.P. (2017). Advanced maternal age and adverse pregnancy outcomes: A systematic review and meta-analysis. PLoS ONE.

[4] Vounzoulaki E., Khunti K., Abner S.C., Tan B.K., Davies M.J., Gillies C.L. (2020). Progression to type 2 diabetes in women with a known history of gestational diabetes: Systematic review and meta-analysis. BMJ.

[5] Esposito G., Mauri P.A., Cipriani S., Franchi M., Corrao G., Parazzini F. (2022). The role of maternal age on the risk of preterm birth among singletons and multiples: A retrospective cohort study in Lombardy, Norther Italy. BMC Pregnancy Childbirth.

[6] Reddy U.M., Ko C.W., Willinger M. (2006). Maternal age and the risk of stillbirth throughout pregnancy in the United States. Am. J. Obstet. Gynecol..

[7] Richards M.K., Flanagan M.R., Littman A.J., Burke A.K., Callegari L.S. (2016). Primary cesarean section and adverse delivery outcomes among women of very advanced maternal age. J. Perinatol..

[8] Vandekerckhove M., Guignard M., Civadier M.S., Benachi A., Bouyer J. (2021). Impact of maternal age on obstetric and neonatal morbidity: A retrospective cohort study. BMC Pregnancy Childbirth.

[9] https://www.cdc.gov/nchs/data/hestat/maternal-mortality/2021/maternal-mortality-rates-2021.htm.

[10] Hypertension in Pregnancy (2013). Report of the American College of Obstetricians and Gynecologists’ Task Force on Hypertension in Pregnancy. Obstet. Gynecol..

[11] Abalos E., Cuesta C., Grosso A.L., Chou D., Say L. (2013). Global and regional estimates of preeclampsia and eclampsia: A systematic review. Eur. J. Obstet. Gynecol. Reprod. Biol..

[12] Ford N.D., Cox S., Ko J.Y., Ouyang L., Romero L., Colarusso T., Ferre C.D., Kroelinger C.D., Hayes D.K., Barfield W.D. (2022). Hypertensive Disorders in Pregnancy and Mortality at Delivery Hospitalization—United States, 2017–2019. MMWR Morb. Mortal. Wkly. Rep..

[13] Magee L.A., Brown M.A., Hall D.R., Gupte S., Hennessy A., Karumanchi S.A., Kenny L.C., McCarthy F., Myers J., Poon L.C. (2022). The 2021 International Society for the Study of Hypertension in Pregnancy classification, diagnosis & management recommendations for international practice. Pregnancy Hypertens..

[14] Khong T.Y., De Wolf F., Robertson W.B., Brosens I. (1986). Inadequate maternal vascular response to placentation in pregnancies complicated by pre-eclampsia and by small-for-gestational age infants. BJOG Int. J. Obstet. Gynaecol..

[15] Maynard S.E., Min J.Y., Merchan J., Lim K.H., Li J., Mondal S., Libermann T.A., Morgan J.P., Sellke F.W., Stillman I.E. (2003). Excess placental soluble fms-like tyrosine kinase 1 (sFlt1) may contribute to endothelial dysfunction, hypertension, and proteinuria in preeclampsia. J. Clin. Investig..

[16] Melchiorre K., Giorgione V., Thilaganathan B. (2022). The placenta and preeclampsia: Villain or victim?. Am. J. Obstet. Gynecol..

[17] Drenthen W., Boersma E., Balci A., Moons P., Roos-Hesselink J.W., Mulder B.J., Vliegen H.W., van Dijk A.P., Voors A.A., Yap S.C. (2010). Predictors of pregnancy complications in women with congenital heart disease. Eur. Heart J..

[18] Foo F.L., Mahendru A.A., Masini G., Fraser A., Cacciatore S., MacIntyre D.A., McEniery C.M., Wilkinson I.B., Bennett P.R., Lees C.C. (2018). Association Between Prepregnancy Cardiovascular Function and Subsequent Preeclampsia or Fetal Growth Restriction. Hypertension.

[19] Keukens A., van Wely M., van der Meulen C., Mochtar M.H. (2022). Pre-eclampsia in pregnancies resulting from oocyte donation, natural conception or IVF: A systematic review and meta-analysis. Hum. Reprod..

[20] Einarsson J.I., Sangi-Haghpeykar H., Gardner M.O. (2003). Sperm exposure and development of preeclampsia. Am. J. Obstet. Gynecol..

[21] Timofeev J., Reddy U.M., Huang C.C., Driggers R.W., Landy H.J., Laughon S.K. (2013). Obstetric complications, neonatal morbidity, and indications for cesarean delivery by maternal age. Obstet. Gynecol..

[22] Bartsch E., Medcalf K.E., Park A.L., Ray J.G., High Risk of Pre-Eclampsia Identification Group (2016). Clinical risk factors for pre-eclampsia determined in early pregnancy: Systematic review and meta-analysis of large cohort studies. BMJ.

[23] Khalil A., Syngelaki A., Maiz N., Zinevich Y., Nicolaides K.H. (2013). Maternal age and adverse pregnancy outcome: A cohort study. Ultrasound Obstet. Gynecol..

[24] Claramonte Nieto M., Meler Barrabes E., Garcia Martínez S., Gutiérrez Prat M., Serra Zantop B. (2019). Impact of aging on obstetric outcomes: Defining advanced maternal age in Barcelona. BMC Pregnancy Childbirth.

[25] Poon L.C., Kametas N.A., Chelemen T., Leal A., Nicolaides K.H. (2010). Maternal risk factors for hypertensive disorders in pregnancy: A multivariate approach. J. Hum. Hypertens..

[26] Lisonkova S., Potts J., Muraca G.M., Razaz N., Sabr Y., Chan W.S., Kramer M.S. (2017). Maternal age and severe maternal morbidity: A population-based retrospective cohort study. PLoS Med..

[27] Smithson S.D., Greene N.H., Esakoff T.F. (2022). Pregnancy outcomes in very advanced maternal age women. Am. J. Obstet. Gynecol. MFM.

[28] Schwartz A., Many A., Shapira U., Rosenberg Friedman M., Yogev Y., Avnon T., Agrawal S., Shinar S. (2020). Perinatal outcomes of pregnancy in the fifth decade and beyond—Acomparison of very advanced maternal age groups. Sci. Rep..

[29] Perry H., Khalil A., Thilaganathan B. (2018). Preeclampsia and the cardiovascular system: An update. Trends Cardiovasc. Med..

[30] Seals D.R., Jablonski K.L., Donato A.J. (2011). Aging and vascular endothelial function in humans. Clin. Sci..

[31] Pirhonen J., Bergersen T.K., Abdlenoor M., Dubiel M., Gudmundsson S. (2005). Effect of maternal age on uterine flow impedance. J. Clin. Ultrasound.

[32] Zalud I., Shaha S. (2008). Three-dimensional sonography of the placental and uterine spiral vasculature: Influence of maternal age and parity. J. Clin. Ultrasound.

[33] Odame Anto E., Owiredu W.K.B.A., Sakyi S.A., Turpin C.A., Ephraim R.K.D., Fondjo L.A., Obirikorang C., Adua E., Acheampong E. (2018). Adverse pregnancy outcomes and imbalance in angiogenic growth mediators and oxidative stress biomarkers is associated with advanced maternal age births: A prospective cohort study in Ghana. PLoS ONE.

[34] Lean S.C., Heazell A.E.P., Dilworth M.R., Mills T.A., Jones R.L. (2017). Placental Dysfunction Underlies Increased Risk of Fetal Growth Restriction and Stillbirth in Advanced Maternal Age Women. Sci. Rep..

[35] Eddy A.C., Howell J.A., Chapman H., Taylor E., Mahdi F., George E.M., Bidwell G.L. (2020). Biopolymer-Delivered, Maternally Sequestered NF-κB (Nuclear Factor-κB) Inhibitory Peptide for Treatment of Preeclampsia. Hypertension.

[36] Wan J., Hu Z., Zeng K., Yin Y., Zhao M., Chen M., Chen Q. (2018). The reduction in circulating levels of estrogen and progesterone in women with preeclampsia. Pregnancy Hypertens..

[37] Ganer Herman H., Volodarsky-Perel A., Ton Nu T.N., Machado-Gedeon A., Cui Y., Shaul J., Dahan M.H. (2023). Diminished ovarian reserve is a risk factor for preeclampsia and placental malperfusion lesions. Fertil. Steril..

[38] de Kat A.C., Verschuren W.M.M., Eijkemans M.J.C., van der Schouw Y.T., Broekmans F.J.M. (2016). The association of low ovarian reserve with cardiovascular disease risk: A cross-sectional population-based study. Hum. Reprod..

[39] Pandey S., Shetty A., Hamilton M., Bhattacharya S., Maheshwari A. (2012). Obstetric and perinatal outcomes in singleton pregnancies resulting from IVF/ICSI: A systematic review and meta-analysis. Hum. Reprod. Update.

[40] Thomopoulos C., Tsioufis C., Michalopoulou H., Makris T., Papademetriou V., Stefanadis C. (2013). Assisted reproductive technology and pregnancy-related hypertensive complications: A systematic review. J. Hum. Hypertens..

[41] Aagaard-Tillery K.M., Silver R., Dalton J. (2006). Immunology of normal pregnancy. Semin. Fetal Neonatal Med..

[42] Imudia A.N., Awonuga A.O., Doyle J.O., Kaimal A.J., Wright D.L., Toth T.L., Styer A.K. (2012). Peak serum estradiol level during controlled ovarian hyperstimulation is associated with increased risk of small for gestational age and preeclampsia in singleton pregnancies after in vitro fertilization. Fertil. Steril..

[43] Chih H.J., Elias F.T., Gaudet L.S., Velez M.P. (2021). Assisted reproductive technology and hypertensive disorders of pregnancy: Systematic review and meta-analyses. BMC Pregnancy Childbirth.

[44] von Versen-Höynck F., Schaub A.M., Chi Y.Y., Chiu K.H., Liu J., Lingis M., Stan Williams R., Rhoton-Vlasak A., Nichols W.W., Fleischmann R.R. (2019). Increased Preeclampsia Risk and Reduced Aortic Compliance with In Vitro Fertilization Cycles in the Absence of a Corpus Luteum. Hypertension.

[45] Chen P., Hu K.-L., Jin J., Chen R., Xu Q., Zhao W., Zhang R., Xing L., Zhu Y., Zhang D. (2022). Risk factors for twin pregnancy in women undergoing double cleavage embryo transfer. BMC Pregnancy Childbirth.

[46] Martin J.A., Osterman M.J.K. (2019). Is Twin Childbearing on the Decline? Twin Births in the United States, 2014–2018. NCHS Data Brief..

[47] Day M.C., Barton J.R., O’Brien J.M., Istwan N.B., Sibai B.M. (2005). The effect of fetal number on the development of hypertensive conditions of pregnancy. Obstet. Gynecol..

[48] Bergman L., Nordlöf-Callbo P., Wikström A.K., Snowden J.M., Hesselman S., Edstedt Bonamy A.K., Sandström A. (2020). Multi-Fetal Pregnancy, Preeclampsia, and Long-Term Cardiovascular Disease. Hypertension.

[49] Bodnar L.M., Ness R.B., Markovic N., Roberts J.M. (2005). The risk of preeclampsia rises with increasing prepregnancy body mass index. Ann. Epidemiol..

[50] Rey E., Couturier A. (1994). The prognosis of pregnancy in women with chronic hypertension. Am. J. Obstet. Gynecol..

[51] Cleary-Goldman J., Malone F.D., Vidaver J., Ball R.H., Nyberg D.A., Comstock C.H., Saade G.R., Eddleman K.A., Klugman S., Dugoff L. (2005). Impact of maternal age on obstetric outcome. Obstet. Gynecol..

[52] Freid V.M., Bernstein A.B., Bush M.A. (2012). Multiple chronic conditions among adults aged 45 and over: Trends over the past 10 years. NCHS Data Brief.

[53] Ogunwole S.M., Mwinnyaa G., Wang X., Hong X., Henderson J., Bennett W.L. (2021). Preeclampsia across Pregnancies and Associated Risk Factors: Findings from a High-Risk US Birth Cohort. J. Am. Heart Assoc..

[54] Hiersch L., Berger H., McDonald S.D., Murray-Davis B., Abdulaziz K.E., Geary M., Barrett J., Melamed N., for DOH-NET (Diabetes, Obesity, and Hypertension in Pregnancy Research Network) and SOON (Southern Ontario Obstetrical Network) Investigators^TM^ (2023). Maternal age and pregnancy outcomes in twin compared with singleton gestations. Int. J. Gynaecol. Obstet..

[55] Whelton P.K., Carey R.M., Aronow W.S., Casey D.E., Collins K.J., Dennison Himmelfarb C., DePalma S.M., Gidding S., Jamerson K.A., Jones D.W. (2018). 2017 ACC/AHA/AAPA/ABC/ACPM/AGS/APhA/ASH/ASPC/NMA/PCNA Guideline for the Prevention, Detection, Evaluation, and Management of High Blood Pressure in Adults: Executive Summary: A Report of the American College of Cardiology/American Heart Association Task Force on Clinical Practice Guidelines. Hypertension.

[56] American College of Obstetricians and Gynecologists’ Committee on Practice Bulletins—Obstetrics (2019). ACOG Practice Bulletin No. 203: Chronic Hypertension in Pregnancy. Obstet. Gynecol..

[57] Tita A.T., Szychowski J.M., Boggess K., Dugoff L., Sibai B., Lawrence K., Hughes B.L., Bell J., Aagaard K., Edwards R.K. (2022). Treatment for Mild Chronic Hypertension during Pregnancy. N. Engl. J. Med..

[58] ACOG Practice Advisory Clinical Guidance for the Integration of the Findings of the Chronic Hypertension and Pregnancy (CHAP) Study. https://www.acog.org/clinical/clinical-guidance/practice-advisory/articles/2022/04/clinical-guidance-for-the-integration-of-the-findings-of-the-chronic-hypertension-and-pregnancy-chap-study.

[59] Clarke R.J., Mayo G., Price P., FitzGerald G.A. (1991). Suppression of thromboxane A2 but not of systemic prostacyclin by controlled-release aspirin. N. Engl. J. Med..

[60] Reddy M., Rolnik D.L., Harris K., Li W., Mol B.W., Da Silva Costa F., Wallace E.M., Palmer K. (2020). Challenging the definition of hypertension in pregnancy: A retrospective cohort study. Am. J. Obstet. Gynecol..

[61] Duley L., Meher S., Hunter K.E., Seidler A.L., Askie L.M. (2019). Antiplatelet agents for preventing pre-eclampsia and its complications. Cochrane Database Syst. Rev..

[62] Rolnik D.L., Wright D., Poon L.C., O’Gorman N., Syngelaki A., de Paco Matallana C., Akolekar R., Cicero S., Janga D., Singh M. (2017). Aspirin versus Placebo in Pregnancies at High Risk for Preterm Preeclampsia. N. Engl. J. Med..

[63] Woo Kinshella M.L., Sarr C., Sandhu A., Bone J.N., Vidler M., Moore S.E., Elango R., Cormick G., Belizan J.M., Hofmeyr G.J. (2022). Calcium for pre-eclampsia prevention: A systematic review and network meta-analysis to guide personalised antenatal care. BJOG.

[64] Brownfoot F.C., Hastie R., Hannan N.J., Cannon P., Tuohey L., Parry L.J., Senadheera S., Illanes S.E., Kaitu’u-Lino T.J., Tong S. (2016). Metformin as a prevention and treatment for preeclampsia: Effects on soluble fms-like tyrosine kinase 1 and soluble endoglin secretion and endothelial dysfunction. Am. J. Obstet. Gynecol..

[65] Kalafat E., Sukur Y.E., Abdi A., Thilaganathan B., Khalil A. (2018). Metformin for prevention of hypertensive disorders of pregnancy in women with gestational diabetes or obesity: Systematic review and meta-analysis of randomized trials. Ultrasound Obstet. Gynecol..

[66] Feng Y., Yang H. (2017). Metformin—A potentially effective drug for gestational diabetes mellitus: A systematic review and meta-analysis. J. Matern. Neonatal Med..

[67] Alqudah A., McKinley M.C., McNally R., Graham U., Watson C.J., Lyons T.J., McClements L. (2018). Risk of pre-eclampsia in women taking metformin: A systematic review and meta-analysis. Diabet. Med..

[68] Syngelaki A., Nicolaides K.H., Balani J., Hyer S., Akolekar R., Kotecha R., Pastides A., Shehata H. (2016). Metformin versus Placebo in Obese Pregnant Women without Diabetes Mellitus. N. Engl. J. Med..

[69] Dodd J.M., Grivell R.M., Deussen A.R., Hague W.M. (2018). Metformin for women who are overweight or obese during pregnancy for improving maternal and infant outcomes. Cochrane Database Syst. Rev..

[70] Løvvik T.S., Carlsen S.M., Salvesen Ø., Steffensen B., Bixo M., Gómez-Real F., Lønnebotn M., Hestvold K.V., Zabielska R., Hirschberg A.L. (2019). Use of metformin to treat pregnant women with polycystic ovary syndrome (PregMet2): A randomised, double-blind, placebo-controlled trial. Lancet Diabetes Endocrinol..

[71] Cluver C., Walker S.P., Mol B.W., Hall D., Hiscock R., Brownfoot F.C., Kaitu’u-Lino T.J., Tong S. (2019). A double blind, randomised, placebo-controlled trial to evaluate the efficacy of metformin to treat preterm pre-eclampsia (PI2 Trial): Study protocol. BMJ Open.

[72] Ahmed A., Ramma W. (2015). Unravelling the theories of pre-eclampsia: Are the protective pathways the new paradigm?. Br. J. Pharmacol..

[73] Costantine M.M., West H., Wisner K.L., Caritis S., Clark S., Venkataramanan R., Stika C.S., Rytting E., Wang X., Ahmed M.S. (2021). A randomized pilot clinical trial of pravastatin versus placebo in pregnant patients at high risk of preeclampsia. Am. J. Obstet. Gynecol..

[74] Kupferminc M.J., Kliger C., Rimon E., Asher-Landsberg J., Skornick-Rapaport A., Gamzu R., Yogev Y. (2022). Pravastatin is useful for prevention of recurrent severe placenta-mediated complications—A pilot study. J. Matern. Neonatal Med..

[75] Akbar M.I.A., Yosediputra A., Pratama R.E., Fadhilah N.L., Sulistyowati S., Amani F.Z., Ernawati E., Dachlan E.G., Angsar M.D., Dekker G. (2021). INOVASIA Study: A Randomized Open Controlled Trial to Evaluate Pravastatin to Prevent Preeclampsia and Its Effects on sFlt1/PlGF Levels. Am. J. Perinatol..

[76] LeFevre M.L., U.S. Preventive Services Task Force (2014). Low-dose aspirin use for the prevention of morbidity and mortality from preeclampsia: U.S. Preventive Services Task Force recommendation statement. Ann. Intern. Med..

[77] Yogev Y., Melamed N., Bardin R., Tenenbaum-Gavish K., Ben-Shitrit G., Ben-Haroush A. (2010). Pregnancy outcome at extremely advanced maternal age. Am. J. Obstet. Gynecol..

[78] Fitzpatrick K.E., Tuffnell D., Kurinczuk J.J., Knight M. (2017). Pregnancy at very advanced maternal age: A UK population-based cohort study. BJOG Int. J. Obstet. Gynaecol..

[79] van der Tuuk K., Koopmans C.M., Groen H., Aarnoudse J.G., van den Berg P.P., van Beek J.J., Copraij F.J., Kleiverda G., Porath M., Rijnders R.J. (2011). Prediction of progression to a high risk situation in women with gestational hypertension or mild pre-eclampsia at term. Aust. N. Z. J. Obstet. Gynaecol..

[80] Ram M., Anteby M., Weiniger C.F., Havakuk O., Gilboa I., Shenhav M., Yogev Y. (2021). Acute pulmonary edema due to severe preeclampsia in advanced maternal age women. Pregnancy Hypertens..

[81] Rymer-Haskel N., Schushan-Eisen I., Hass Y., Rahav R., Maayan-Metzger A., Hendler I. (2018). Characteristics and severity of preeclampsia in young and elderly gravidas with hypertensive disease. Eur. J. Obstet. Gynecol. Reprod. Biol..

[82] Cooke C.M., Davidge S.T. (2019). Advanced maternal age and the impact on maternal and offspring cardiovascular health. Am. J. Physiol. Circ. Physiol..

[83] Walters B.N., Walters T. (1987). Hypertension in the puerperium. Lancet.

[84] Peterson E., Craigo S., House M. (2010). Risk factors for postpartum antihypertensive medication requirement in severe preeclampsia. Hypertens. Pregnancy.

[85] Sibai B.M. (2012). Etiology and management of postpartum hypertension-preeclampsia. Am. J. Obstet. Gynecol..

[86] Makris A., Thornton C., Hennessy A. (2004). Postpartum hypertension and nonsteroidal analgesia. Am. J. Obstet. Gynecol..

[87] Stott D., Papastefanou I., Paraschiv D., Clark K., Kametas N.A. (2017). Serial hemodynamic monitoring to guide treatment of maternal hypertension leads to reduction in severe hypertension. Ultrasound Obstet. Gynecol..

[88] Levine R.J., Maynard S.E., Qian C., Lim K.H., England L.J., Yu K.F., Schisterman E.F., Thadhani R., Sachs B.P., Epstein F.H. (2004). Circulating angiogenic factors and the risk of preeclampsia. N. Engl. J. Med..

[89] Verlohren S., Herraiz I., Lapaire O., Schlembach D., Zeisler H., Calda P., Sabria J., Markfeld-Erol F., Galindo A., Schoofs K. (2014). New gestational phase-specific cutoff values for the use of the soluble fms-like tyrosine kinase-1/placental growth factor ratio as a diagnostic test for preeclampsia. Hypertension.

[90] Zeisler H., Llurba E., Chantraine F., Vatish M., Staff A.C., Sennström M., Olovsson M., Brennecke S.P., Stepan H., Allegranza D. (2016). Predictive Value of the sFlt-1:PlGF Ratio in Women with Suspected Preeclampsia. N. Engl. J. Med..

[91] Rana S., Powe C.E., Salahuddin S., Verlohren S., Perschel F.H., Levine R.J., Lim K.H., Wenger J.B., Thadhani R., Karumanchi S.A. (2012). Angiogenic factors and the risk of adverse outcomes in women with suspected preeclampsia. Circulation.

[92] German Society of Gynaecology and Obstetrics, Austrian Society for Gynecology and Obstetrics, Swiss Society for Gynecology and Obstetrics Hypertensive Pregnancy Disorders: Diagnosis and Therapy. Guideline of the German Society of Gynecology and Obstetrics (S2k-Level, AWMF-Registry No. 015/018, March 2019) 2018. https://www.awmf.org/.

[93] Rolfo A., Attini R., Nuzzo A.M., Piazzese A., Parisi S., Ferraresi M., Todros T., Piccoli G.B. (2013). Chronic kidney disease may be differentially diagnosed from preeclampsia by serum biomarkers. Kidney Int..

[94] Kim M.Y., Buyon J.P., Guerra M.M., Rana S., Zhang D., Laskin C.A., Petri M., Lockshin M.D., Sammaritano L.R., Branch D.W. (2015). Angiogenic factor imbalance early in pregnancy predicts adverse outcomes in patients with lupus and antiphospholipid antibodies: Results of the Promisse study. Am. J. Obstet. Gynecol..

[95] Cohen A.L., Wenger J.B., James-Todd T., Lamparello B.M., Halprin E., Serdy S., Fan S., Horowitz G.L., Lim K.H., Rana S. (2014). The association of circulating angiogenic factors and HbA1c with the risk of preeclampsia in women with preexisting diabetes. Hypertens. Pregnancy.

[96] Crovetto F., Figueras F., Triunfo S., Crispi F., Rodriguez-Sureda V., Dominguez C., Llurba E., Gratacós E. (2015). First trimester screening for early and late preeclampsia based on maternal characteristics, biophysical parameters, and angiogenic factors. Prenat. Diagn..

[97] Verlohren S., Brennecke S.P., Galindo A., Karumanchi S.A., Mirkovic L.B., Schlembach D., Stepan H., Vatish M., Zeisler H., Rana S. (2022). Clinical interpretation and implementation of the sFlt-1/PlGF ratio in the prediction, diagnosis and management of preeclampsia. Pregnancy Hypertens..

[98] Soundararajan R., Suresh S.C., Mueller A., Heimberger S., Avula S., Sathyanarayana C., Mahesh S., Madhuprakash S., Rana S. (2021). Real life outpatient biomarker use in management of hypertensive pregnancies in third trimester in a low resource SeTting: ROBUST study. Pregnancy Hypertens..

[99] ACOG (2019). Practice Bulletin No. 202: Gestational Hypertension and Preeclampsia. Obstet. Gynecol..

[100] Koopmans C.M., Bijlenga D., Groen H., Vijgen S.M., Aarnoudse J.G., Bekedam D.J., van den Berg P.P., de Boer K., Burggraaff J.M., Bloemenkamp K.W. (2009). Induction of labour versus expectant monitoring for gestational hypertension or mild pre-eclampsia after 36 weeks’ gestation (HYPITAT): A multicentre, open-label randomised controlled trial. Lancet.

[101] Brown M.C., Best K.E., Pearce M.S., Waugh J., Robson S.C., Bell R. (2013). Cardiovascular disease risk in women with pre-eclampsia: Systematic review and meta-analysis. Eur. J. Epidemiol..

